# Seeding of Dermal Substitutes with Glucose-Pretreated Nanofat Accelerates In Vivo Vascularization and Tissue Integration

**DOI:** 10.3390/jfb16090311

**Published:** 2025-08-28

**Authors:** Valeria Pruzzo, Francesca Bonomi, Ettore Limido, Andrea Weinzierl, Yves Harder, Matthias W. Laschke

**Affiliations:** 1 Institute for Clinical and Experimental Surgery, Saarland University, PharmaScienceHub (PSH), 66421 Homburg, Germany; pruzzovaleria@gmail.com (V.P.); francescabonomi.bonnie@gmail.com (F.B.); limidoettore@gmail.com (E.L.); andrea.weinzierl@icloud.com (A.W.); 2 Department of Surgery, Ospedale Beata Vergine Mendrisio, Ente Ospedaliero Cantonale (EOC), 6850 Mendrisio, Switzerland; 3 Department of Plastic Surgery and Hand Surgery, University Hospital Zurich, 8006 Zurich, Switzerland; 4 Department of Plastic, Reconstructive and Aesthetic Surgery and Hand Surgery, Centre Hospitalier Universitaire Vaudois (CHUV), 1005 Lausanne, Switzerland; yves.harder@yahoo.com; 5 Faculty of Biology and Medicine, University of Lausanne (UNIL), 1005 Lausanne, Switzerland

**Keywords:** angiogenesis, dermal substitutes, glucose, nanofat, skin defect

## Abstract

The exposure of endothelial cells to high glucose concentrations promotes angiogenesis. The present study investigated whether this pro-angiogenic effect of glucose is suitable to improve the capability of nanofat to vascularize implanted dermal substitutes. Nanofat was processed from white adipose tissue originating from green fluorescent protein (GFP)^+^ C57BL/6J donor mice and incubated for 1 h in Hank’s Balanced Salt Solution with or without (control) a high level of glucose (30 mM). The pretreated nanofat was seeded onto dermal substitutes, which were analyzed by intravital fluorescence microscopy, histology and immunohistochemistry in dorsal skinfold chambers of GFP^−^ C57BL/6J mice to assess their vivo performance over a period of 14 days. A high level of glucose-pretreated nanofat did not induce a stronger immune response when compared to the control. However, it improved the vascularization of the implants, as shown by a significantly higher density of blood-perfused microvessels in the border zones (~3.6-fold increase) and more CD31^+^/GFP^+^ microvessels (~3-fold increase) inside the implants. Accordingly, high glucose-pretreated nanofat levels also enhanced the tissue integration of the dermal substitutes, as indicated by the deposition of more type I collagen (~2.9-fold increase). These findings suggest that the short-term exposure of nanofat to a high level of glucose represents a promising and clinically feasible strategy to enhance its regenerative properties when seeded onto dermal substitutes.

## 1. Introduction

The management of full-thickness skin defects resulting from burns, trauma, tumor excision or metabolic disorders is a significant challenge in the field of plastic and reconstructive surgery, particularly if the blood flow to the tissue is impaired or soft tissue is diseased [1]. When the extent or quality of the wound bed precludes immediate autologous skin grafting, a multi-stage surgical process is required. In this case, initial wound coverage with a porous dermal substitute provides a scaffold for dermal regeneration and reconstruction [2,3,4]. Successful final coverage using split-thickness skin grafts requires sufficient blood perfusion and the proper integration of the implanted dermal substitute into the host tissue. However, this is often a time-consuming process, which hinders the timely reconstruction of the physiological skin barrier and, eventually, exposes patients to an increased risk of infections [5].

To ensure the rapid and sufficient vascularization of dermal substitutes, various approaches have been tested [6]. These include seeding the implants with purified components of adipose tissue, including adipose-derived stem cells (ASCs), endothelial cells and microvascular fragments [5,7]. However, the isolation of these components is usually achieved by the enzymatic digestion of fat samples and may also include additional cell culture steps, which represents a substantial tissue manipulation and, thus, raises safety concerns when clinically applied [8,9]. In contrast, nanofat is an autologous fat derivative that can be rapidly obtained through the mechanical emulsification and filtration of adipose tissue without any further manipulation even during surgery [10]. Moreover, it represents a mixture of ASCs, microvascular fragments, growth factors and extracellular matrix components that may synergistically promote microvascular network formation and tissue regeneration [11]. Due to these properties and its fluid consistency, nanofat is considered to be a versatile option for a broad clinical application spectrum comprising soft tissue rejuvenation, wound management, scar remodeling, peripheral nerve regeneration as well as the treatment of joint degenerative diseases and alopecia [12,13,14].

Recently, nanofat is increasingly used in tissue engineering, particularly with the aim of enhancing blood perfusion and the tissue incorporation of bone grafts and dermal substitutes [15,16,17]. However, even nanofat-seeded dermal substitutes still exhibit a rather late onset of vascularization [16,17]. To overcome this problem, nanofat may be pretreated with a suitable compound before seeding it onto dermal substitutes to stimulate its angiogenic activity. In our study, we speculated that highly concentrated glucose (30 mM) may be a suitable option. In fact, high glucose levels are known to induce aberrant angiogenesis in diabetic patients, which leads to complications such as proliferative retinopathy, nephropathy and neuropathy [18,19,20]. In addition, several preclinical studies focusing on pathological angiogenesis induced by diabetes reported that endothelial cells cultured under high-glucose conditions (~30 mM) exhibit a significantly increased proliferation, migration and tube formation capacity [21,22,23,24,25,26]. Additionally, in contrast to the use of pro-angiogenic recombinant growth factors, the use of glucose represents a cost-effective and, from a regulatory point of view, harmless approach to stimulate nanofat.

Based on these findings, we, herein, shortly incubated freshly generated nanofat from transgenic green fluorescent protein (GFP)^+^ donor mice for 1 h in Hank’s Balanced Salt Solution (HBSS) supplemented with or without a high level of glucose (30 mM). Subsequently, the pretreated nanofat was seeded onto dermal substitutes, which were analyzed in dorsal skinfold chambers of immunocompetent GFP^−^ recipient mice to assess their in vivo performance, as previously performed [16,17].

## 2. Materials and Methods

### 2.1. Mice

The animal study protocol was approved by the local authorities (permission number: 19-2024; State Office for Consumer Protection, Saarbrücken, Germany, 9 October 2024). All animal experiments were performed according to the ARRIVE guidelines, the European legislation on the protection of animals (Directive 2010/63/EU) and the National Institutes of Health (NIH) Guidelines on the Care and Use of Laboratory Animals (NIH publication #85-23 Rev. 1985).

To generate nanofat, white adipose tissue was harvested from the groin of 8 male and female C57BL/6-Tg (CAG-EGFP)131Osb/LeySopJ mice (age: ~9 months; weight: >30 g; The Jackson Laboratory, Bar Harbor, ME, USA). The use of this GFP^+^ strain allowed the distinction between GFP^+^ nanofat-derived cells and GFP^−^ cells of the surrounding host tissue. Of note, male and female donors were mixed because we demonstrated in a previous study that the sex of the donors does not markedly determine the in vivo vascularization capacity of nanofat [11]. In addition, 16 C57BL/6J mice (age: ~5 months; weight ~22–30 g; both sexes) received a dorsal skinfold chamber. The animals were housed under standard laboratory conditions with free access to water and pellet chow (ssniff Spezialdiäten GmbH, Soest, Germany) over the entire experimental period. They were obtained from the Institute for Clinical and Experimental Surgery (Saarland University, Homburg, Germany) or Charles River Laboratories (Sulzfeld, Germany).

### 2.2. Anesthesia

For all surgical interventions and microscopic analyses, the mice were anesthetized by intraperitoneally injected ketamine hydrochloride (100 mg/kg body weight; Ketabel^®^; Bela-pharm GmbH & Co. KG, Vechta, Germany) combined with xylazine (12 mg/kg body weight; Rompun^®^; Bayer, Leverkusen, Germany). To treat perioperative pain, carprofen (10 mg/kg body weight; Rimadyl^®^; Zoetis Deutschland GmbH, Berlin, Germany) was injected subcutaneously. Ophthalmic ointment served for the prevention of dry eyes (Bepanthen^®^; Bayer Vital GmbH, Leverkusen, Germany).

### 2.3. Processing and Pretreatment of Nanofat

White adipose tissue harvested from donor mice was mechanically processed into nanofat according to established protocols [11,16]. For this purpose, the adipose tissue was washed in physiological saline solution and cut in smaller samples with a dimension of ~1 mm × 1 mm × 1 mm (McIlwain Tissue Chopper, CLE Co. Ltd., Gomshall, UK). The samples were repeatedly bidirectionally pressed through three female-to-female Luer lock connectors (diameter: 2.4, 1.4 and 1.2 mm) with two syringes (30 times per connector). The suspension then underwent a final filtration step using a 500 µm filter to remove larger tissue fragments. The obtained nanofat was equally divided into two tubes (Eppendorf, Hamburg, Germany) and incubated for 1 h at a 1:1 volume ratio with either HBSS (Gibco, Waltham, MA, USA) alone as the vehicle (control, n = 8) or HBSS supplemented with 30 mM glucose (n = 8; VWR International, Leuven, Belgium). This high glucose concentration has previously been shown to be effective and well tolerated in animal experiments [22].

### 2.4. Preparation and Seeding of Dermal Substitutes

Clinically approved dermal substitutes (Integra^®^; Integra LifeSciences, Ghent, Belgium) with a thickness of 1.3 mm were cut into circular samples (Ø 4 mm) using a sterile tissue punch (Kai Europe GmbH, Solingen, Germany). Each sample was incubated for 10 min at room temperature in the tubes containing vehicle- or glucose-pretreated nanofat for seeding. This incubation period enabled the adhesion of nanofat on the porous sample, as previously proven by histological analysis [16].

### 2.5. Animal Model and Microscopic Analysis

Dermal substitutes seeded with vehicle- or glucose-pretreated nanofat were implanted into the observation window of dorsal skinfold chambers in GFP^−^ mice [16]. In combination with intravital fluorescence microscopy, this model enables non-invasive, in vivo analyses of blood vessel formation within implants, while preventing implant dehydration or animal automanipulation [27].

Stereomicroscopic images of the dermal substitutes were repeatedly taken over 14 days under a Leica M651 stereomicroscope (Leica, Wetzlar, Germany). At identical time points, i.e., day 0 (implantation day), 3, 6, 10 and 14, intravital fluorescence microscopic imaging was additionally performed. Prior to each microscopy session, 50 µL of 5% fluorescein isothiocyanate (FITC)-labeled dextran (150,000 Da; Sigma-Aldrich, Taufkirchen, Germany) for plasma staining and 50 µL of 0.1% rhodamine 6G (Sigma-Aldrich) for leukocyte staining were intravenously injected in the plexus behind the eye [16].

Repeated intravital fluorescence microscopy was conducted using a Zeiss Axiotech fluorescence epi-illumination microscope (Zeiss, Oberkochen, Germany) with an Axiocam 702 mono camera (Carl Zeiss Microscopy GmbH, Oberkochen, Germany). Subsequent image analysis was performed with CapImage (version 8.10.1; Dr. Zeintl Software, Heidelberg, Germany). The dermal substitutes were analyzed in 8 regions of interest (ROIs) located in the border (n = 4) and center (n = 4) of the implants. The total number of perfused ROIs, defined by the presence of newly formed red blood cell (RBC)-perfused microvessels, was assessed (given in %). Additionally, the total length of the RBC-perfused microvessels within each ROI was measured to determine the functional microvessel density (given in cm/cm^2^). Furthermore, five microvessels within each ROI were randomly selected in order to measure their diameter (given in µm) and centerline RBC velocity (given in µm/s). These measurements served for the subsequent calculation of the shear rate (given in s^−1^) and volumetric blood flow (given in pL/s).

The inflammatory response was evaluated in 4 peri-implant postcapillary and collecting venules by assessing microhemodynamic parameters and the interaction of leukocytes with the microvascular endothelium. Leukocytes were categorized as free-flowing, rolling or adherent cells. Rolling leukocytes (given in min^−1^) were identified by a reduced velocity and repeated interaction with the microvascular endothelium. Adherent leukocytes (given in mm^−2^ of the endothelial surface) exhibited a firm attachment to the endothelium for a period of 30 s. The endothelial surface area was calculated assuming a cylindrical vessel architecture.

### 2.6. Histology and Immunohistochemistry

After the last in vivo microscopy, the deeply anesthetized mice were euthanized by cervical dislocation. The dorsal skin containing the dermal substitutes was harvested and further processed for different stainings of tissue sections. These stainings included hematoxylin and eosin (HE) as well as the immunohistochemical detection of CD31, lymphatic vessel endothelial hyaluronan receptor (LYVE)-1, collagen (Col) I, Col III, CD68, myeloperoxidase (MPO) and CD3 [16].

Quantitative analyses of the stained sections were performed using a BX53 microscope (Olympus, Hamburg, Germany) and the cellSens Dimension software (version 1.11; Olympus, Hamburg, Germany). The density (mm^−2^) of the CD31^+^ microvessels and LYVE-1^+^ lymphatic vessels was evaluated in both the border and center zones of each implant by dividing the total number of vessels by the ROI area. The proportion of GFP^+^ blood and lymphatic vessels (expressed as the percentage of all vessels) was also quantified in both groups. Additionally, the ratio of the total Col I and Col III, relative to normal skin, was determined. Immune cell infiltration was assessed by quantifying CD68^+^ macrophages, MPO^+^ neutrophilic granulocytes and CD3^+^ lymphocytes (given in mm^−2^), based on the analysis of two ROIs in the center and two ROIs in the border zone of each implant.

### 2.7. Statistical Analysis

The normal distribution and equal variance of the assessed data were checked with GraphPad Prism (version 10.1.2; GraphPad Software, San Diego, CA, USA). Thereafter, group comparisons were performed by an unpaired Student’s t-test (parametric data) or Mann-Whitney rank-sum test (non-parametric data). The results are presented as mean ± standard error of the mean (SEM), with the statistical significance defined as *p* < 0.05.

## 3. Results

### 3.1. In Vivo Microscopy

**Figure 1:**
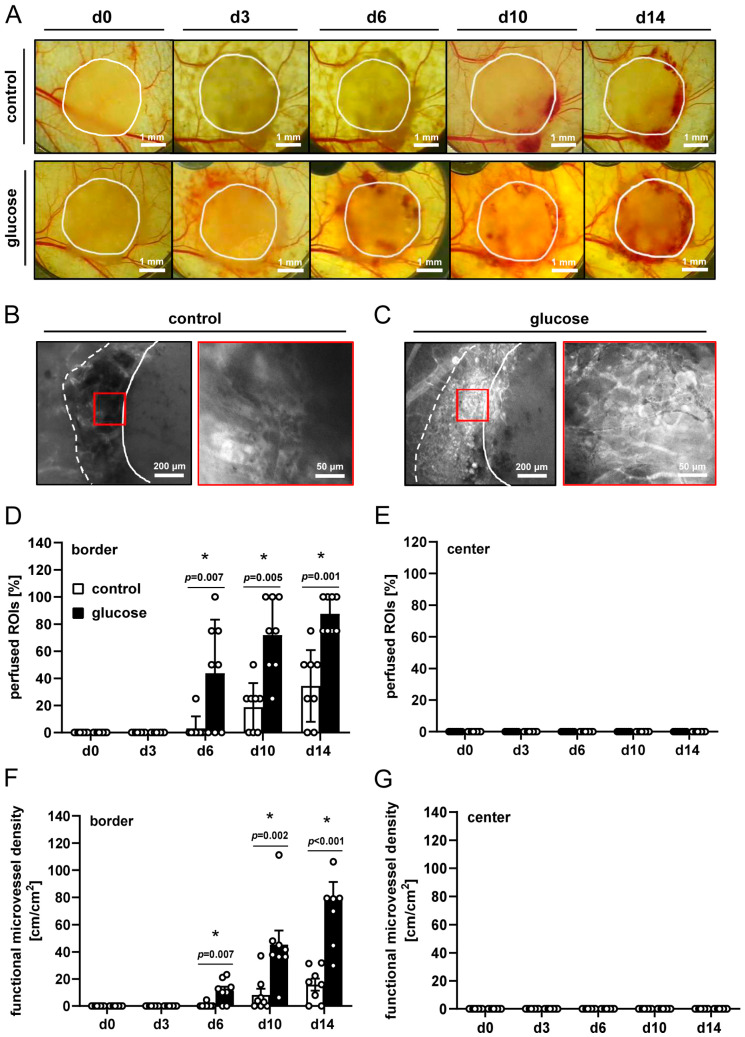
In vivo microscopy. (**A**) Representative stereomicroscopic images of dermal substitutes seeded with vehicle-pretreated nanofat (control, upper panels) or glucose-pretreated nanofat (glucose, lower panels) on days 0–14 (closed line indicates implant border). (**B**,**C**) Representative intravital fluorescence microscopic images of dermal substitutes seeded with vehicle-pretreated nanofat (control, (**B**)) or glucose-pretreated nanofat (glucose, (**C**)) on day 14 (closed line indicates implant border; broken line indicates border of vascularized area; red frame indicates regions of interest (ROIs) in the border of the implants shown on the right panel in higher magnification). (**D**–**G**) Perfused ROIs (%) (**D**,**E**) and functional microvessel density (cm/cm^2^) (**F**,**G**) in the border (**D**,**F**) and center zones (**E**,**G**) of dermal substitutes seeded with vehicle-pretreated nanofat (control; white bars, n = 8) and glucose-pretreated nanofat (glucose; black bars, n = 8) on days 0–14 post-implantation, as analyzed by intravital fluorescence microscopy. Mean ± SEM. * *p* < 0.05 vs. control.

Dermal substitutes seeded with vehicle- or glucose-pretreated nanofat showed different host tissue reactions during repeated stereomicroscopic imaging throughout the experimental period of 2 weeks (Figure 1A). Of note, dorsal skinfold chambers containing dermal substitutes seeded with glucose-pretreated nanofat exhibited more extensive tissue bleedings, as indicated by the red-colored tissue areas within and around the implants between days 3 and 14 as a sign of a pronounced angiogenic response to the implants (Figure 1A).

Intravital fluorescence microscopy revealed the development of a new microvasculature along the borders of the dermal substitutes (Figure 1B,C). This process was accelerated for dermal substitutes seeded with glucose-pretreated nanofat, as shown by a higher number of perfused ROIs and the functional microvessel density in the border zones of the implants between day 6 and 14 when compared to the controls (Figure 1D,F). On the other hand, no microvessels were observed in the center of the dermal substitutes in both groups (Figure 1E,G). Moreover, the microhemodynamic parameters of individual microvessels were comparable in both groups (Table 1). However, in contrast to the control group, these parameters could already be assessed on day 6 for dermal substitutes seeded with glucose-pretreated nanofat (Table 1), which further indicates an accelerated vascularization process.

**Figure 2:**
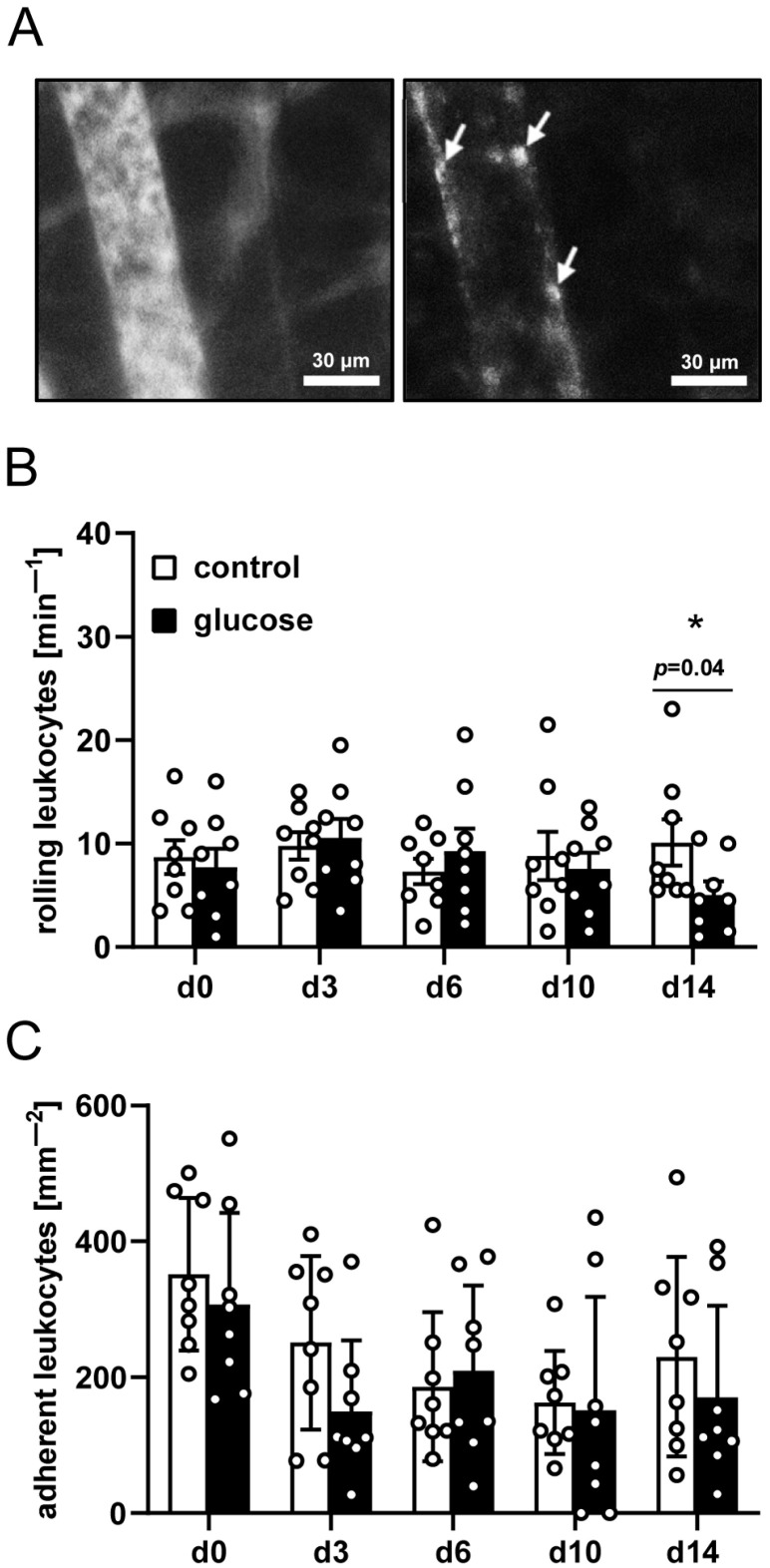
Leukocyte-endothelial cell interaction. (**A**) Representative intravital fluorescence microscopic images of a collecting venule next to a dermal substitute seeded with vehicle-pretreated nanofat (blue light epi-illumination, contrast enhancement by 5% FITC-labeled dextran (left panel); green light epi-illumination, in situ staining of leukocytes with 0.1% rhodamine 6G (right panel); arrows indicate leukocytes). (**B**,**C**) Rolling leukocytes (min^−1^) (**B**) and adherent leukocytes (mm^−2^) (**C**) within postcapillary and collecting venules next to dermal substitutes seeded with vehicle-pretreated nanofat (control; white bars, n = 8) and glucose-pretreated nanofat (black bars, n = 8) on days 0–14 post-implantation, as analyzed by intravital fluorescence microscopy. Mean ± SEM. * *p* < 0.05 vs. control.

To evaluate the immune response to the dermal substitutes, interactions of individual leukocytes with the endothelium of peri-implant postcapillary and collecting venules were assessed. For this purpose, the leukocytes were stained in situ with 0.1% rhodamine 6G to visualize them in green light epi-illumination (Figure 2A). The venules were visualized in blue light epi-illumination after the injection of the plasma marker 5% fluorescein isothiocyanate (FITC)-labeled dextran (Figure 2A). In both groups, these vessels did not differ in terms of microhemodynamic parameters (Table 2). Furthermore, the numbers of rolling and adherent leukocytes were comparable in the two groups (Figure 2B,C).

### 3.2. Histological and Immunohistochemical Evaluation

**Figure 3:**
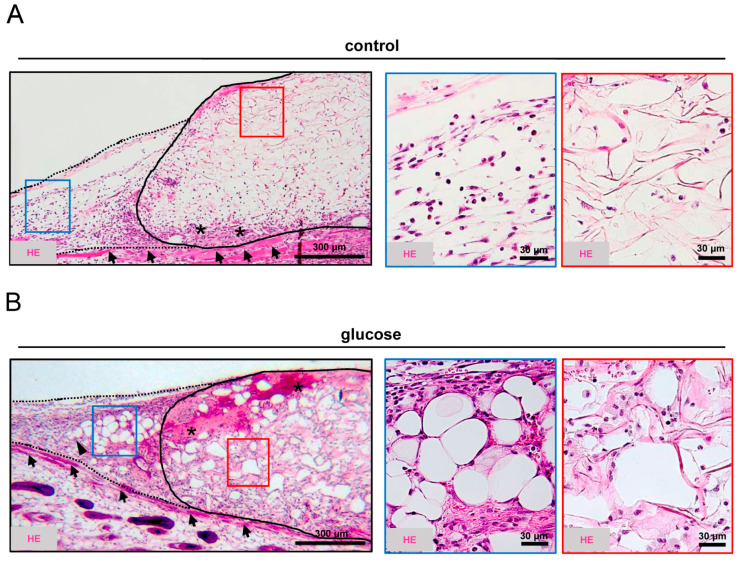
Tissue integration. (**A**,**B**) Representative HE-stained sections of dermal substitutes seeded with vehicle-pretreated nanofat (control, (**A**)) or glucose-pretreated nanofat (glucose, (**B**)) on day 14 post-implantation within dorsal skinfold chambers of C57BL/6J recipient mice (closed line indicates implant border; broken line indicates border zone; blue and red frames indicate ROIs in the border and center zones of the implants shown in higher magnification on the left panels; arrows indicate panniculus carnosus muscle; asterisks indicate granulation tissue; arrowhead indicates adipocytes).

After the in vivo experiments, the dermal substitutes were additionally evaluated by histology and immunohistochemistry. These analyses revealed that the dermal substitutes seeded with glucose-pretreated nanofat were better incorporated into the surrounding tissue compared to the controls (Figure 3A,B). In fact, the granulation tissue in their border zones exhibited a higher cellular density and still residual adipocytes originating from the seeded nanofat (Figure 3B). Throughout the 14-day implantation period, this granulation tissue had also grown into the dermal substitutes, finally filling up many pores of the implants (Figure 3B).

**Figure 4:**
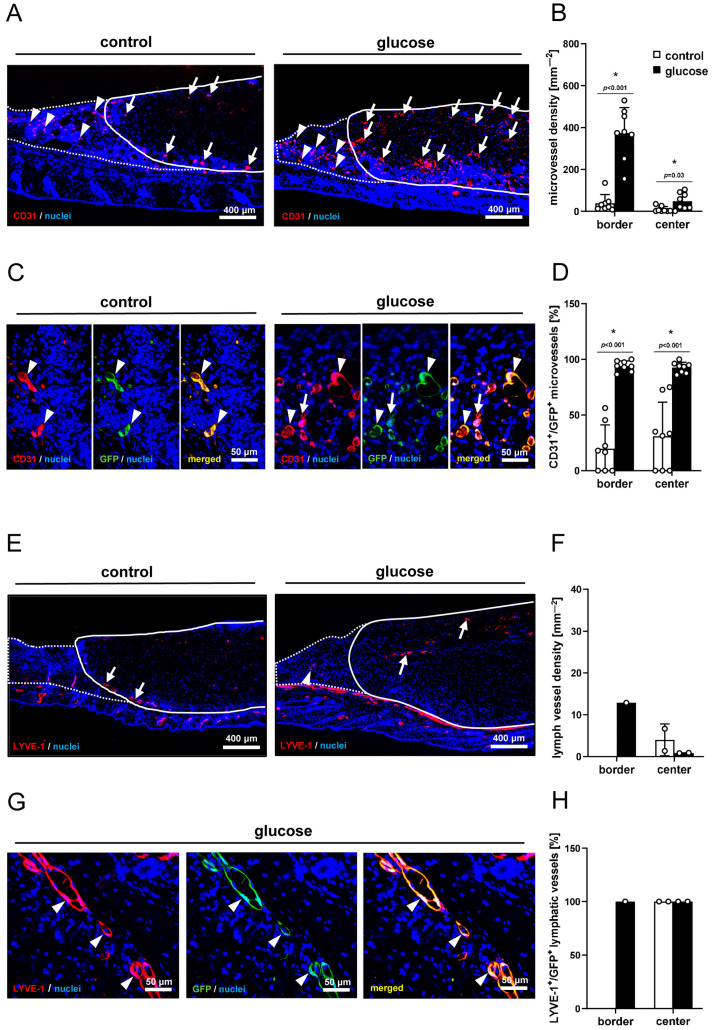
Vascularization and lymph vessels. (**A**) Representative immunohistochemical sections showing CD31^+^ microvessels in the border zones (arrowheads) and center zones (arrows) of dermal substitutes seeded with vehicle-pretreated nanofat (control) or glucose-pretreated nanofat on day 14 (closed line indicates implant border; broken line indicates border zones). (**B**) Microvessel density (mm^−2^) of dermal substitutes seeded with vehicle-pretreated nanofat (control; white bars, n = 8) and glucose-pretreated nanofat (glucose; black bars, n = 8) on day 14, as analyzed by immunohistochemistry. Mean ± SEM. * *p* < 0.05 vs. control. (**C**) Representative immunohistochemical sections showing CD31^+^/GFP^−^ (arrows) and CD31^+^/GFP^+^ (arrowheads) microvessels in nanofat-seeded dermal substitutes on day 14. (**D**) CD31^+^/GFP^+^ microvessels (%) in the border zones and center zones of dermal substitutes seeded with vehicle-pretreated nanofat (control; white bars, n = 8) and glucose-pretreated nanofat (glucose; black bars, n = 8) on day 14, as analyzed by immunohistochemistry. Mean ± SEM. * *p* < 0.05 vs. control. (**E**) Representative immunohistochemical sections showing LYVE-1^+^ lymph vessels in the border zones (arrowhead) and center zones (arrows) of dermal substitutes seeded with vehicle-pretreated nanofat (control) or glucose-pretreated nanofat (glucose) on day 14 (closed line indicates implant border; broken line indicates border zones). (**F**) Lymph vessel density (mm^−2^) of dermal substitutes seeded with vehicle-treated nanofat (control; white bars, n = 2) and glucose-pretreated nanofat (glucose; black bars, n = 1–2) on day 14, as analyzed by immunohistochemistry. Mean ± SEM. (**G**) Representative immunohistochemical sections showing LYVE-1^+^/GFP^+^ (arrowheads) lymph vessels in dermal substitutes seeded with vehicle-pretreated nanofat on day 14. (**H**) LYVE-1^+^/GFP^+^ microvessels (%) in the border and center zones of dermal substitutes seeded with vehicle-pretreated nanofat (control; white bars, n = 2) and glucose-pretreated nanofat (glucose; black bars, n = 1–2) on day 14, as analyzed by immunohistochemistry. Mean ± SEM.

The immunohistochemical CD31 stainings for the detection of microvessels demonstrated that dermal substitutes seeded with glucose-pretreated nanofat had a 12.6-fold higher and 5-fold higher microvessel density in their border and center zones, respectively (Figure 4A,B). Additional CD31/GFP co-stainings showed that more than 90% of the microvessels within the dermal substitutes seeded with glucose-pretreated nanofat expressed GFP. This showed that they originated from the seeded GFP^+^ nanofat (Figure 4C,D). In contrast, this fraction was markedly lower in dermal substitutes seeded with vehicle-pretreated nanofat (Figure 4D). In addition, the immunohistochemical detection of the lymph vessel marker LYVE-1 revealed that there were only very few lymphatic vessels in the border and center zones of the dermal substitutes without significant differences between the two groups (Figure 4E,F). Of note, all of these lymph vessels expressed GFP (Figure 4G,H).

**Figure 5:**
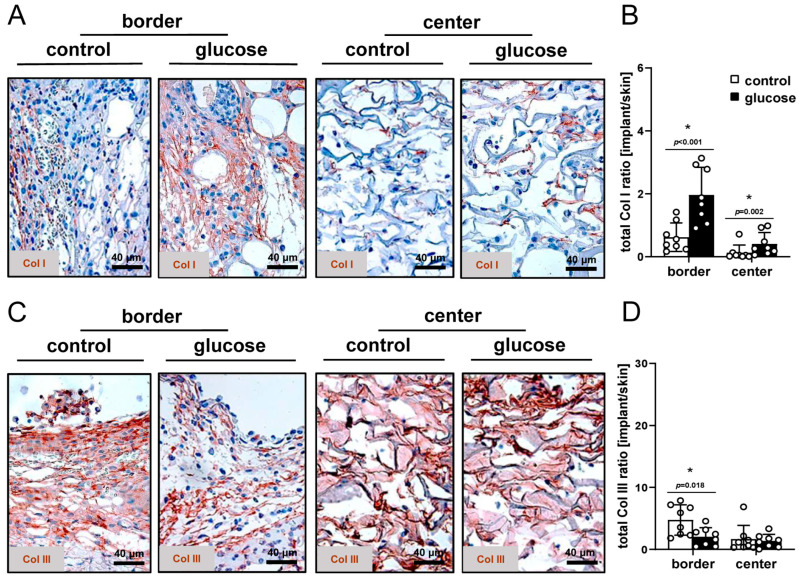
Col content. (**A**,**C**) Representative immunohistochemical sections showing Col I (**A**) and III (**C**) in the border and center zones of dermal substitutes seeded with vehicle-pretreated nanofat (control) or glucose-pretreated nanofat on day 14. (**B**,**D**) Total Col I (**B**) and Col III (**D**) ratio (implant/skin) in the border and center zones of dermal substitutes seeded with vehicle-pretreated nanofat (control; white bars, n = 8) and glucose-pretreated nanofat (glucose; black bars, n = 8) on day 14, as analyzed by immunohistochemistry. Mean ± SEM. * *p* < 0.05 vs. control.

To investigate the tissue integration of the implants in more detail, the Col content of the dermal substitutes was additionally quantified while differentiating between Col I and III. Dermal substitutes seeded with glucose-pretreated nanofat showed a significantly higher total Col I ratio in the border and center zones and a significantly lower Col III ratio at the borders (Figure 5A–D).

**Figure 6:**
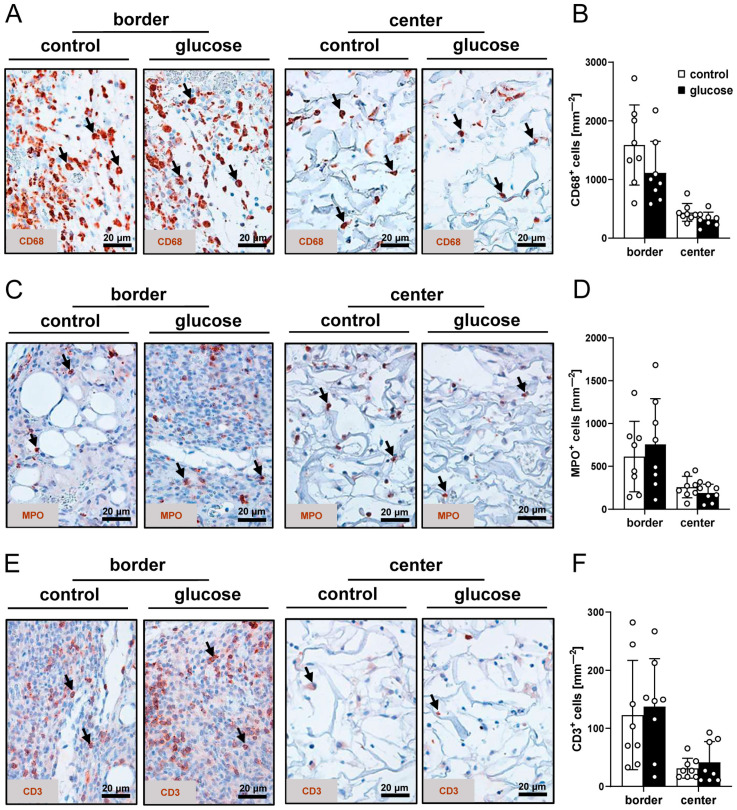
Infiltration of immune cells. (**A**,**C**,**E**) Representative immunohistochemical sections showing CD68^+^ macrophages (**A**, arrows), MPO^+^ granulocytes (**C**, arrows) and CD3^+^ lymphocytes (**E**, arrows) in the border and center zones of dermal substitutes seeded with vehicle-pretreated nanofat (control) or glucose-pretreated nanofat (glucose) on day 14. (**B**,**D**,**F**) CD68^+^ macrophages (mm^−2^) (**B**), MPO^+^ granulocytes (mm^−2^) (**D**) and CD3^+^ lymphocytes (mm^−2^) (**F**) in the border and center zones of dermal substitutes seeded with vehicle-pretreated nanofat (control; white bars, n = 8) and glucose-pretreated nanofat (glucose; black bars, n = 8) on day 14, as analyzed by immunohistochemistry. Mean ± SEM.

Finally, the infiltration of immune cells into the implants was evaluated by means of immunohistochemical sections stained against the macrophage marker CD68, the neutrophilic granulocyte marker MPO and the lymphocyte marker CD3. There were no significant differences between the two implant types (Figure 6A–F).

## 4. Discussion

Delayed or insufficient blood perfusion is a major challenge during the clinical application of dermal substitutes [28]. To overcome this problem, a clinically applicable and effective approach remains to be established [29]. Our study provides the first proof-of-concept that the short-term pretreatment of nanofat with a high level of glucose prior to seeding onto dermal substitutes can accelerate their vascularization and integration into the wound bed, thereby potentially reducing the overall time required for final skin reconstruction.

Over the years, multiple studies have attributed the pro-angiogenic effect of glucose to the overexpression of angiogenic growth factors [18,30]. Among these, vascular endothelial growth factor (VEGF) is a key angiogenic cytokine that promotes the proliferation and migration of endothelial cells and increases the microvascular permeability [31]. On the other hand, nanofat contains large numbers of ASCs with the capability of differentiating into endothelial cells and, thus, fostering tissue vascularization [32]. Therefore, it can be assumed that the combined action of glucose and nanofat is a potent stimulus for the formation of a new microvasculature.

Furthermore, nanofat contains numerous intact microvascular fragments. It is well known that these fragments can interconnect with each other into functional microvascular networks and also develop anastomoses with vessels of the host tissue via inosculation [33,34]. Of note, we previously demonstrated that this process is enhanced by exposure of isolated microvascular fragments to high glucose concentrations (30 mM) for 24 h under culture conditions [35]. In the present study, we wanted to take advantage of this beneficial effect of high glucose while simultaneously establishing a protocol that could easily be implemented into clinical practice. Accordingly, nanofat was incubated at a 1:1 volume ratio with either HBSS or a high level of glucose at room temperature for only 1 h, which would make the intraoperative pretreatment of nanofat feasible. In this context, it should be noted that others reported that the storage of adipose tissue at room temperature up to 8 h does not change its viability and biological properties [36,37]. From a clinical perspective, this targeted ex situ short-term exposure of nanofat to high levels of glucose may be particularly attractive as it prevents potential side effects that may be associated with the systemic administration of glucose. Importantly, this easy and short intervention was still highly effective, as indicated throughout our in vivo analyses. In fact, we found that dermal substitutes seeded with glucose-pretreated nanofat exhibited a significantly higher number of blood-perfused ROIs and a higher functional microvessel density between day 6 and 14 after implantation, indicating the accelerated vascularization of the implants over time.

In accordance with our in vivo observations, immunohistochemical analyses of the dermal substitutes seeded with glucose-pretreated nanofat showed a markedly higher microvessel density and fraction of CD31^+^/GFP^+^ microvessels when compared to the controls. The latter result indicates that substantially more microvessels originating from nanofat survived in the high glucose group and/or exhibited higher proliferating activity. Moreover, implants seeded with glucose-pretreated nanofat contained more adipocytes, suggesting the enhanced survival of these cells following pretreatment with high levels of glucose. This view is supported by previous studies reporting that the glucose exposure of ASCs promotes a degree of differentiation in mature adipocytes and lipid accumulation by increasing the expression of adipogenesis-regulating genes [38].

An early inflammatory response is crucial for initiating physiological tissue regeneration [39]. However, persistent or excessive inflammation is known to impair this process [39,40]. Therefore, the immune response to the implants was additionally investigated in the present study. We found that leukocyte-endothelial cell interactions in venules of the host tissue and the immune cell infiltration of the implants did not markedly differ between dermal substitutes seeded with vehicle- or glucose-pretreated nanofat. Thus, we conclude that both implant types exhibit a comparable biocompatibility. Moreover, the accelerated and improved vascularization of dermal substitutes seeded with glucose-pretreated nanofat was not caused by the induction of inflammation, which is known to be a strong stimulator of angiogenesis [41,42,43].

The tissue integration of the nanofat-seeded dermal substitutes was additionally assessed by the immunohistochemical detection of Col deposition within the border and center zones of the implants. Interestingly, dermal substitutes seeded with glucose-pretreated nanofat presented with a significantly higher content of Col I in both regions and a reduced presence of Col III in the border zones compared to the controls. It is well known that the newly developing granulation tissue in early wound healing is typically rich in Col III, whereas later maturation and remodeling phases are characterized by a predominance of Col I [44]. Transferred to our own findings, this indicates that dermal substitutes seeded with glucose-pretreated nanofat exhibited more mature granulation tissue on day 14 and, thus, better tissue integration into the host tissue. This improved tissue integration can be explained by the accelerated vascularization of dermal substitutes seeded with glucose-pretreated nanofat. In fact, the proliferation of endothelial cells and subsequent angiogenesis is essential for the synthesis, deposition and organization of a new collagen network [45].

Dermal substitutes may also better integrate into the surrounding tissue by the development of a draining lymphatic system [16]. Previous studies have shown that nanofat is also a source of lymphatic vessel fragments, which are able to survive in the granulation tissue and integrate into new lymphatic networks, possibly increasing fluid drainage [46]. Therefore, the effect of glucose exposure on the presence of lymphatic vessels within the nanofat-seeded dermal substitutes was also investigated in the present study. However, only very few LYVE-1^+^/GFP^+^ lymph vessels were found in the border and center zones of some implants with no significant differences between the two groups. Hence, we assume that lymphangiogenesis is not a mechanism that substantially contributed to the observed differences in tissue integration between dermal substitutes seeded with vehicle- and glucose-pretreated nanofat.

Finally, this study also faces some limitations. The maximum observation period in the dorsal skinfold chamber model is usually not longer than 14 days. Hence, it was not possible to analyze the implants until complete vascularization and tissue integration. However, our main interest was to clarify whether the glucose pretreatment of nanofat accelerates these processes, which could be ideally clarified in this model in the early phase after the implantation of the dermal substitutes. Nonetheless, further studies should additionally clarify the long-term effects of our intervention, particularly on fibrosis and scarring. Furthermore, we used healthy donor and recipient animals in our study. However, especially diabetic ulcers represent a large fraction of non-healing wounds, which may benefit from future nanofat-based therapies. Therefore, it would be also interesting to investigate the therapeutic outcome and safety of our approach in diabetic models. In addition, it must be clarified whether the herein observed positive effects of glucose pretreatment are reproducible for nanofat of a human origin.

In conclusion, this study provides the first evidence that the short-term exposure of nanofat to high levels of glucose improves its angiogenic potential. Accordingly, dermal substitutes seeded with glucose-pretreated nanofat exhibit accelerated vascularization and tissue integration after implantation. However, their biocompatibility is not affected by this intervention. The herein described short-term ex vivo stimulation of nanofat with high glucose levels could be easily implemented in an intraoperative setting. Hence, it may not only be a promising approach to promote skin reconstruction by means of nanofat-seeded dermal substitutes, but also to improve the regenerative capacity of nanofat for other applications, such as peripheral nerve regeneration. Therefore, this approach now requires further testing in appropriate clinical trials. This is not only necessary to confirm its efficiency under clinical conditions, which may be much more challenging due to differing co-morbidities and medications of patients, but also to prove its feasibility and safety.

## Figures and Tables

**Table 1:** Diameter (µm), centerline red blood cell (RBC) velocity (µm/s), shear rate (s^−1^) and volumetric blood flow (pL/s) of microvessels within the border and center zones of dermal substitutes seeded with vehicle-pretreated nanofat (control; n = 8) and glucose-pretreated nanofat (glucose; n = 8) on days 0–14 post-implantation, as analyzed by intravital fluorescence microscopy. Mean ± SEM. No significant differences.

	d0	d3	d6	d10	d14
***diameter* (µm):**					
border: control	-	-	-	14.4 ± 1.1	16.7 ± 0.9
glucose	-	-	9.8 ± 0.8	17.0 ± 1.3	18.3 ± 0.8
center: control	-	-	-	-	-
glucose	-	-	-	-	-
***centerline RBC velocity* (µm/s):**					
border: control	-	-	-	169.1 ± 28.3	239.3 ± 23.0
glucose	-	-	24.8 ± 13	167.2 ± 39.5	207.3 ± 28.8
center: control	-	-	-	-	-
glucose	-	-	-	-	-
***shear rate* (s^−1^):**					
border: control	-	-	-	109.8 ± 26.6	125.1 ± 13.5
glucose	-	-	52.8 ± 7.2	96.9 ± 21.1	97.9 ± 13.1
center: control	-	-	-	-	-
glucose	-	-	-	-	-
***volumetric blood flow* (pL/s):**					
border: control	-	-	-	16.1 ± 2.8	36.5 ± 7.5
glucose	-	-	3.5 ± 1.3	27.6 ± 7.9	31.7 ± 5.7
center: control	-	-	-	-	-
glucose	-	-	-	-	-

**Table 2:** Diameter (µm), centerline RBC velocity (µm/s), shear rate (s^−1^) and volumetric blood flow (pL/s) of postcapillary and collecting venules next to dermal substitutes seeded with vehicle-pretreated nanofat (control; n = 8) and glucose-pretreated nanofat (glucose; n = 8) on days 0–14 post-implantation, as analyzed by intravital fluorescence microscopy. Mean ± SEM. No significant differences.

	d0	d3	d6	d10	d14
***diameter* (µm):**					
control	37.2 ± 2.2	36.7 ± 1.3	35.2 ± 1.1	35.0 ± 1.9	33.5 ± 0.9
glucose	34.1 ± 1.3	35.7 ± 0.8	38.2 ± 0.9	34.2 ± 1.4	32.7 ± 0.9
***centerline RBC velocity* (µm/s):**					
control	330.0 ± 36.6	367.1 ± 22.4	372.4 ± 30.5	262.8 ± 28.7	325.6 ± 22.0
glucose	275.9 ± 19.1	325.3 ± 23.9	318.4 ± 20.3	291.3 ± 25.0	291.9 ± 20.0
***shear rate* (s** **^−1^):**					
control	72.3 ± 8.0	80.6 ± 4.8	86.2 ± 7.0	61.0 ± 6.0	79.6 ± 4.8
glucose	61.1 ± 5.9	74.5 ± 5.4	68.2 ± 3.8	68.9 ± 5.6	72.7 ± 6.8
***volumetric blood flow* (pL/s):**					
control	239.0 ± 42.1	259.6 ± 23.4	237.9 ± 29.8	169.1 ± 34.1	184.7 ± 20.5
glucose	193.7 ± 12.1	208.0 ± 21.0	239.8 ± 24.1	175.2 ± 22.7	171.0 ± 11.5

## Data Availability

The original contributions presented in this study are included in the article and further inquiries can be directed to the corresponding author.

## Abbreviations

This paper contains these abbreviations:

ASCs	Adipose-derived stem cells
Col	Collagen
FITC	Fluorescein isothiocyanate
GFP	Green fluorescent protein
HBSS	Hank’s Balanced Salt Solution
HE	Hematoxylin and eosin
LYVE	Lymphatic vessel endothelial hyaluronan receptor
RBC	Red blood cell
ROIs	Regions of interest
SEM	Standard error of the mean
